# Eucommia ulmoides extract and soy isoflavones attenuate postmenopausal osteoporosis by modulating bone—specific transcription factors and inflammatory signaling pathways

**DOI:** 10.3389/fphar.2026.1753846

**Published:** 2026-05-08

**Authors:** Ruihong Zhao, Jinhuan He, Mengdi Wang, Yiming Li, Shiyu Hao, Yiying Chen, Bao Liu, Yabo Liu, Guoqiang Wang

**Affiliations:** 1 College of Food and Biological Engineering, Henan University of Animal Husbandry and Economy, Zhengzhou, China; 2 Department of Respiratory and Critical Care Medicine, Henan Provincial People’s Hospital, Zhengzhou, China; 3 Academy of Chinese Medical Sciences, Henan University of Chinese Medicine, Zhengzhou, China

**Keywords:** bone remodeling, Eucommia ulmoides, inflammatory signaling, postmenopausal osteoporosis, soy isoflavones, transcriptional regulation

## Abstract

**Background:**

Postmenopausal osteoporosis (PMO) is driven by estrogen deficiency, enhanced osteoclastic activity, impaired osteoblast function, and persistent inflammatory signaling. Eucommia ulmoides extract (EUE) and soy isoflavones (Isf) are known to possess phytoestrogenic and anti—inflammatory properties, but their combined molecular effects on PMO remain unclear.

**Methods:**

An ovariectomy—induced PMO rat model was used to assess the therapeutic efficacy of EUE, Isf, and their combination. Bone strength and mineral content were evaluated together with serum indicators of bone turnover, including type I procollagen N—terminal propeptide, alkaline phosphatase, C—terminal telopeptide of type I collagen, and tartrate—resistant acid phosphatase. Serum calcium, phosphorus, superoxide dismutase, and malondialdehyde levels were also measured. Trabecular microarchitecture was assessed histologically. *In vitro*, osteoblast differentiation was examined by Alizarin Red staining and proliferative activity by EdU incorporation, while osteoclast formation and apoptosis were analyzed by tartrate—resistant acid phosphatase staining and flow cytometry. Mechanistic evaluation focused on bone—related transcriptional regulators such as Runx2 and alkaline phosphatase, key mediators of osteoclastogenesis including RANKL, RANK, OPG, and TRAP, and inflammatory cytokines including tumor necrosis factor—α, interleukin—6, interleukin—1β, and interleukin—10.

**Results:**

The combined EUE and Isf treatment significantly improved biomechanical performance of the femur and restored bone mineral content in ovariectomized rats. Levels of bone formation markers were elevated, whereas indices of bone resorption were reduced. Serum calcium–phosphorus homeostasis and oxidative stress were markedly corrected, accompanied by substantial improvement in trabecular structure. *In vitro*, the combination enhanced osteoblast mineralization and proliferation, suppressed osteoclast formation, and increased osteoclast apoptosis. Mechanistically, the combined treatment increased the expression of osteogenic regulators Runx2, alkaline phosphatase, and osteoprotegerin, while reducing the expression of RANKL, RANK, and TRAP. Pro—inflammatory cytokines were lowered, whereas interleukin—10 was upregulated.

**Conclusion:**

EUE combined with soy isoflavones effectively attenuates postmenopausal osteoporosis by enhancing osteoblast function, suppressing osteoclastogenesis, and rebalancing bone—related transcriptional and inflammatory pathways. This botanical combination represents a promising therapeutic strategy for the management of PMO.

## 1 Introduction

Postmenopausal osteoporosis (PMO) is a progressive skeletal disorder characterized by decreased bone mass, deterioration of trabecular structure, and increased fracture risk (Fischer and Haffner-Luntzer, 2022). Estrogen deficiency after menopause disrupts the dynamic coupling between osteoclast—mediated bone resorption and osteoblast—driven bone formation, leading to accelerated bone loss (Zhang et al., 2022). At the molecular level, estrogen withdrawal enhances osteoclast survival, increases osteoclastic resorptive activity, and suppresses osteoblast differentiation through multiple pathways including increased production of tumor necrosis factor—α, interleukin—6, and other inflammatory mediators (Gandhi et al., 2024; Zhang et al., 2025). These inflammatory cytokines further amplify osteoclastogenesis by stimulating the RANKL–RANK axis while simultaneously downregulating osteoblast—related transcription factors such as Runx2 and alkaline phosphatase (Jiang et al., 2023; Wang et al., 2025). Thus, PMO reflects a complex interplay among hormonal imbalance, inflammatory activation, oxidative stress, and impaired transcriptional regulation of bone remodeling (Weitzmann and Pacifici, 2006; Manolagas, 2010; Wang et al., 2023).

Although several pharmacological agents—such as bisphosphonates, selective estrogen receptor modulators, and monoclonal antibodies targeting RANKL—are clinically available, long—term treatment is often limited by adverse effects, high cost, or poor patient adherence (Händel et al., 2023; Kubi et al., 2024). These limitations have driven increasing interest in natural products with dual regulatory effects on osteoblasts and osteoclasts, as well as more favorable safety profiles.

*Eucommia ulmoides*, a traditional medicinal plant widely used in East Asia, is rich in lignans, iridoids, phenolic acids, and flavonoids (Huang Q. et al., 2021). Extracts of *Eucommia ulmoides* demonstrate antioxidant, anti—inflammatory, and phytoestrogen—like effects and have been shown to prevent bone loss in estrogen—deficient models (Feng et al., 2022; Yin et al., 2025). Soy isoflavones, another class of phytoestrogenic compounds, selectively activate estrogen receptor β and exert protective effects on bone by inhibiting osteoclast formation and modulating inflammatory responses (Messina et al., 2025). Despite these known benefits, the combined therapeutic potential of *Eucommia ulmoides* extract (EUE) and soy isoflavones (Isf) in PMO has not been systematically investigated.

Moreover, whether EUE and Isf act synergistically on key regulatory pathways—including bone—specific transcriptional programs and inflammatory signaling networks—and whether such combined modulation translates into improved bone microarchitecture and biomechanical strength remain unanswered questions. Addressing these gaps is crucial for developing evidence—based botanical interventions that target both osteoblast and osteoclast lineages.

In this study, we investigated the therapeutic efficacy and mechanistic basis of EUE combined with Isf in an ovariectomy—induced rat model of PMO and in complementary *in vitro* bone cell systems. We focused on the regulation of transcription factors essential for osteoblast differentiation, such as Runx2 and alkaline phosphatase, and on the modulation of osteoclastogenesis through the RANKL–RANK–OPG signaling axis. Additionally, given the established role of inflammatory cytokines in bone deterioration, we assessed the impact of the combined treatment on tumor necrosis factor—α, interleukin—6, interleukin—1β, and the anti—inflammatory mediator interleukin—10. Through integrating *in vivo* and *in vitro* analyses, this study aims to elucidate whether EUE and Isf exert complementary effects on bone remodeling pathways and to determine their potential as a botanical therapeutic strategy for postmenopausal osteoporosis.

## 2 Materials and methods

### 2.1 Plant materials and preparation of Eucommia ulmoides extract (EUE)

Eucommia ulmoides leaf extract (EUE) was prepared by the manufacturer (Changsha Shanghe Biotechnology Co., Ltd.) using the following method: dried leaves of Eucommia ulmoides were pulverized and extracted with 70% ethanol (v/v) at a solid—to—solvent ratio of 1:10 (w/v) under reflux conditions for 2 h. The extraction was performed twice, and the combined extracts were filtered, concentrated under reduced pressure using a rotary evaporator (50 °C), and spray—dried to obtain a fine powder. The extraction yield was approximately 12–15% (w/w). The plant material was authenticated in accordance with the 2020 edition of the Chinese Pharmacopoeia, and a voucher specimen (Batch No. EUE–20230518) was deposited at the Academy of Chinese Medical Sciences, Henan University of Chinese Medicine. The extract was standardized to contain 30% chlorogenic acid, as determined by high—performance liquid chromatography (HPLC). The chemical profile of EUE was characterized by HPLC fingerprinting using an Agilent 1260 HPLC system equipped with a C18 column (4.6 × 250 mm, 5 μm). The mobile phase consisted of 0.1% phosphoric acid in water (A) and acetonitrile (B) with a gradient elution program: 0–15 min, 10–20% B; 15–30 min, 20–40% B; 30–40 min, 40–60% B. The flow rate was 1.0 mL/min, detection wavelength was 254 nm, and column temperature was maintained at 30 °C. The extract was stored in sealed, light—protected aluminum foil pouches at room temperature until use. For both *in vitro* and *in vivo* studies, EUE was freshly dissolved in sterile 0.9% saline (Kelun Pharmaceutical, Chengdu, China) immediately prior to administration.

### 2.2 Soy isoflavones (Isf)

Soy isoflavones were purchased from Yuanye Biotechnology (Shanghai, China) and standardized to contain 40% total isoflavones, comprising genistein, daidzein, and glycitein. Purity was confirmed by high—performance liquid chromatography (HPLC) using the same chromatographic conditions as previously described. For cell—based experiments, stock solutions were prepared in dimethyl sulfoxide (DMSO, ≥99.9%, Sigma—Aldrich, St. Louis, MO, United States) and subsequently diluted with culture medium to the desired concentrations, ensuring that the final DMSO content did not exceed 0.1%.

### 2.3 Cell culture

The murine osteoblast cell line MC3T3—E1 and the osteoclast precursor cell line RAW264.7 were obtained from the Stem Cell Bank, Chinese Academy of Sciences (Shanghai, China), in accordance with their original characterization. MC3T3—E1 cells were cultured in α—minimum essential medium (α—MEM), and RAW264.7 cells in high—glucose Dulbecco’s modified Eagle’s medium (DMEM) (Gibco, Thermo Fisher Scientific, Waltham, MA, United States), each supplemented with 10% fetal bovine serum (Gibco, United States) and 1% penicillin–streptomycin (Solarbio, Beijing, China). All cells were maintained at 37 °C in a humidified incubator with 5% CO_2_ (Thermo Forma Series II, Thermo Fisher Scientific, United States). Bone marrow mesenchymal stem cells (BMSCs) were isolated according to previously published protocols.

### 2.4 Cell viability assay (MTT)

Cell viability was evaluated using the MTT assay under conditions consistent with those described in the original protocol. MC3T3—E1 cells were seeded in 96—well plates at a density of 5 × 10^3^ cells per well and treated with EUE (10–500 μg/mL) or isoflavones (Isf, 20–320 μg/mL) for 48 h. Subsequently, MTT solution (0.5 mg/mL; Solarbio, Beijing, China) was added to each well and incubated for 4 h. The resulting formazan crystals were solubilized in dimethyl sulfoxide (DMSO; Sigma—Aldrich, United States), and absorbance was measured at 570 nm using a microplate reader (BioTek Synergy H1, Winooski, VT, United States).

### 2.5 Animal model and experimental design

All animal procedures were conducted in accordance with the approval of the Ethics Committee of Henan University of Chinese Medicine (Approval No. IACUC—2023X078). Twelve—week—old female Sprague–Dawley rats were obtained from the Experimental Animal Center of Henan University of Chinese Medicine (Zhengzhou, China) and housed under controlled environmental conditions: temperature (25 °C ± 2 °C), humidity (55% ± 5%), and a 12—hour light/dark cycle.

Bilateral ovariectomy (OVX) was performed under anesthesia with sodium pentobarbital (40 mg/kg; Sinopharm, Shanghai, China). Sham—operated animals underwent the same surgical procedure without removal of the ovaries. After a 2—weeks postoperative recovery period, rats were randomly assigned to six groups: Sham, OVX, OVX + EUE (300 mg/kg), OVX + isoflavones (Isf, 18.8 mg/kg), OVX + EUE + Isf, and OVX + raloxifene (1 mg/kg; Eli Lilly, Indianapolis, IN, United States). The doses of EUE (300 mg/kg/day) and Isf (18.8 mg/kg/day) were selected based on preliminary experiments and previous literature (Liao et al., 2003). All treatments were administered orally once daily for 12 weeks.

### 2.6 Femoral biomechanical testing

Femora were harvested, carefully cleaned of soft tissue, and stored at 4 °C in saline—moistened gauze until analysis. Mechanical strength was assessed using a universal testing machine (Instron 5943, Instron, Norwood, MA, United States) following a three—point bending protocol. Maximum load, elastic modulus, and maximum stress were automatically calculated using Bluehill Universal software (Instron, United States).

### 2.7 Bone mineral and ash content

Femurs were dried at 105 °C in a laboratory oven (Shanghai Yiheng, China) and subsequently ashed in a muffle furnace (Boxun SX2—5–12A, Shanghai, China). The resulting ash was dissolved in nitric acid, and calcium content was determined using inductively coupled plasma optical emission spectrometry (ICP—OES; Agilent 5110, Agilent Technologies, United States).

### 2.8 Serum biochemical analysis and oxidative stress markers

Blood samples were centrifuged at 3,000 rpm for 10 min using a refrigerated centrifuge (Xiangyi H1650R, Hunan, China) to separate serum. Serum levels of procollagen type I N—terminal propeptide (PINP), alkaline phosphatase (ALP), C—terminal telopeptide of type I collagen (CTX—1), tartrate—resistant acid phosphatase (TRAP), superoxide dismutase (SOD), and malondialdehyde (MDA) were quantified using enzyme—linked immunosorbent assay (ELISA) kits (Elabscience, Wuhan, China), and absorbance was measured with a microplate reader (BioTek Synergy H1, United States). Serum calcium and phosphorus concentrations were determined using an automated biochemical analyzer (Mindray BS—240, Shenzhen, China).

### 2.9 Histological examination

Fixed femurs were decalcified in 10% ethylenediaminetetraacetic acid (EDTA) and processed using a tissue processor (Leica TP1020, Germany). Paraffin—embedded sections (5 μm) were prepared using a microtome (Leica RM2235, Germany) and stained with hematoxylin and eosin (H&E; Solarbio, Beijing, China). Histological images were captured using a light microscope (Nikon Eclipse Ci—L, Tokyo, Japan).

### 2.10 Osteoblast differentiation and mineralization

BMSCs were induced with osteogenic differentiation medium and treated with EUE, isoflavones (Isf), or their combination (EUE + Isf) for 21 days. Mineralized nodule formation was assessed by Alizarin Red S staining (Solarbio, China), and quantification was performed using ImageJ software (NIH, United States). Cell proliferation was evaluated using an EdU assay kit (RiboBio, Guangzhou, China), and fluorescence images were captured with a fluorescence microscope (Olympus IX73, Japan).

### 2.11 Osteoclast differentiation and apoptosis

RAW264.7 cells were induced with recombinant murine receptor activator of nuclear factor—κB ligand (RANKL; PeproTech, United States) to differentiate into osteoclasts. Tartrate—resistant acid phosphatase (TRAP) staining was performed using a TRAP staining kit (Sigma—Aldrich, United States). Apoptosis was quantified using an Annexin V—FITC/propidium iodide (PI) apoptosis detection kit (Beyotime, Shanghai, China) and analyzed by flow cytometry (BD FACSCanto II, BD Biosciences, United States).

### 2.12 Quantitative real—time PCR

Total RNA was extracted using an RNA purification kit (Tiangen, Beijing, China) and reverse—transcribed into cDNA using a cDNA synthesis kit (Takara, Shiga, Japan). Quantitative real—time PCR (qPCR) was conducted on a StepOnePlus Real—Time PCR System (Applied Biosystems, United States) with SYBR Green Master Mix (Applied Biosystems, United States). Relative gene expression levels were calculated using the 2^−^ΔΔCt method. Primer information is shown in Table 1.

**TABLE 1:** Primer sequences.

Gene	Forward primer	Reverse primer
ALP	GATAAGGAGGACTTCAAG	CTC​ACT​ACA​CAG​TAA​GAT​AA
RunX2	TTA​GTG​TAA​TGG​TCT​GTT​GA	ATGCCTGAAGGAATTGAA
OPG	AACAGAGAAGCAACTCAA	TTC​GGT​ATA​ATC​TTG​GTA​GG
RANKL	TGGCTTCTATTACCTGTA	TAA​CGA​CAT​ATA​CCA​TCA​G
TRAP	CTAAAGAAATCGCCAGAA	TCCAGTGAAGTAGAAGTT
RANK	TGC​TCC​AGT​AGT​GAT​ATT​C	AGT​GCC​AAA​TAA​TGT​AAA​GG
β-Actin	TAT​GGA​ATC​CTG​TGG​CAT​C	GTG​TTG​GCA​TAG​AGG​TCT​T

### 2.13 Western blotting

Protein concentrations were determined using a BCA protein assay kit (Beyotime, Shanghai, China). Equal amounts of protein were separated by SDS–polyacrylamide gel electrophoresis (SDS—PAGE; Bio—Rad, Hercules, CA, United States) and transferred onto polyvinylidene fluoride (PVDF) membranes (Millipore, United States). After blocking, membranes were incubated with primary antibodies (Cell Signaling Technology, United States), followed by visualization using enhanced chemiluminescence (ECL) reagents (Thermo Fisher Scientific, United States) on a ChemiDoc XRS + imaging system (Bio—Rad, United States). The antibody information is shown in Table 2.

**TABLE 2:** Antibody.

Antibody	Dilution ratio	Number	Company
Anti—TRAP/CD40L antibody [EP462E]	1/5,000	ab52750	Abcam
Anti—RUNX2 antibody [2B9]	1/5,000	ab76956	Abcam
Anti—Osteoprotegerin antibody	1/3,000	ab73400	Abcam
Anti—RANKL antibody	1/3,000	ab9957	Abcam
Beta Actin Monoclonal Antibody (15G5A11/E2)	1/1,000	MA1—140	Invitrog
Anti—IL—6 antibody [EPR23819—11]	1/1,000	ab259341	Abcam
Anti—TNF alpha antibody [EPR19147]	1/1,000	ab183218	Abcam
Anti—IL—1 beta antibody [EPR23851—127]	1/1,000	ab254360	Abcam
Anti—IL—10 antibody [EPR1114]	1/1,000	ab133575	Abcam

### 2.14 Statistical analysis

All data are presented as mean ± standard deviation (SD). Statistical analysis was performed using one—way analysis of variance (ANOVA) followed by Tukey’s *post hoc* test for multiple comparisons. For comparisons between two groups, Student’s t—test was used. —. Graphs were generated using GraphPad Prism version 9.0 (GraphPad Software, United States). A p—value <0.05 was considered statistically significant.

## 3 Results

### 3.1 Effects of Eucommia ulmoides extract (EUE) and soy isoflavones (Isf) on MC3T3—E1 cell viability

**FIGURE 1:**
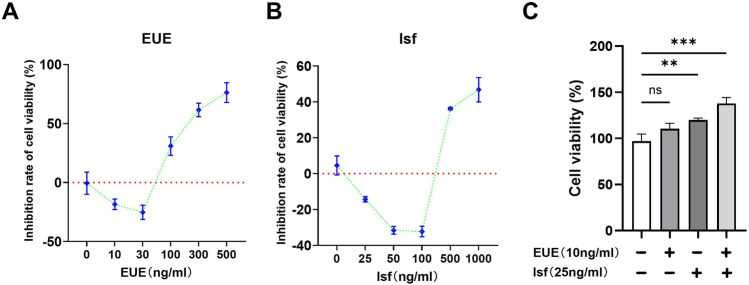
Effects of EUE and Isf on MC3T3—E1 cell viability. **(A)** Dose–response effects of EUE (0–500 μg/mL) on cell viability. **(B)** Dose–response effects of Isf (0–1,000 μg/mL). **(C)** Combined low—dose EUE (10 μg/mL) and Isf (25 μg/mL) synergistically increased cell viability. Data are mean ± SD (n = 3). ns, not significant; **p < 0.01; ***p < 0.001.

As shown in Figure 1A, EUE exhibited a distinct concentration—dependent biphasic effect on MC3T3—E1 cell viability. At low concentrations (10–30 μg/mL), EUE significantly increased cell viability, as reflected by the negative inhibition rate (approximately −10% at 10 ng/mL and −20% at 30 ng/mL), indicating a clear proliferative effect. However, with further increases in concentration, this stimulatory action was reversed. EUE at 300 ng/mL elevated the inhibition rate to nearly 40%, and at 500 ng/mL the inhibition rate exceeded 60%, demonstrating a strong cytotoxic effect at higher doses. This upward shift across the right half of the curve (Figure 1A) confirms that EUE transitions from promoting proliferation at low doses to inducing growth suppression at high doses.

Similarly, Figure 1B shows that Isf also produced a hormetic response. Low—dose Isf (25–50 μg/mL) reduced the inhibition rate to −10% to −30%, indicating enhanced cell viability. When the concentration reached 100 μg/mL, the inhibition rate approached −35%, representing the maximal proliferative window. However, at 500 ng/mL and especially at 1,000 ng/mL, the inhibition rate rapidly rose to approximately 35%–45%, indicating significant cytotoxicity. The sharp upward inflection beyond 100 μg/mL in Figure 1B highlights the dose threshold where beneficial activity shifts to toxicity.

As shown in Figure 1C, the combination of low—dose EUE (10 μg/mL) and Isf (25 μg/mL) produced a markedly greater proliferative effect than either compound alone. Although individual treatments resulted in modest increases in viability (approximately 105%–120%), the combined treatment raised viability to nearly 150%, representing a highly significant synergistic enhancement (*p* < 0.001). The stepwise elevation of the bar height in Figure 1C vividly illustrates this synergistic interaction, confirming that co—administration of EUE and Isf at low concentrations amplifies osteoblast proliferation within the optimal growth—promoting range.

### 3.2 Effects of EUE and Isf on uterine morphology, histology, and femoral biomechanical properties

**FIGURE 2:**
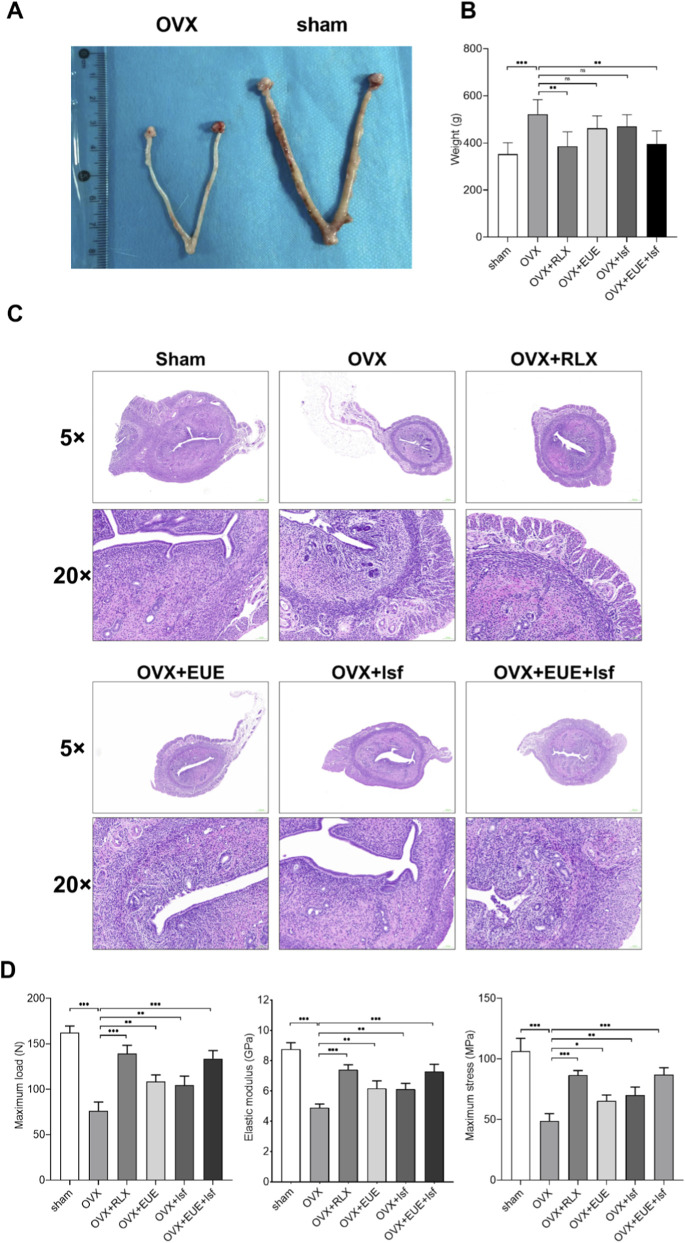
Effects of EUE and Isf on uterine morphology, histology, and femoral biomechanical properties. **(A)** Representative macroscopic images of uteri from each group showing uterine atrophy in OVX rats and partial or marked recovery after treatment. **(B)** Uterine weight showing significant decrease after ovariectomy and improvement following treatment. **(C)** H&E—stained uterine sections (5× and 20×) demonstrating structural atrophy in the OVX group and variable degrees of restoration by raloxifene, EUE, Isf, and combined treatment. **(D)** Femoral biomechanical parameters (maximum load, elastic modulus, and maximum stress) indicating OVX—induced reductions and treatment—related recovery. Data are expressed as mean ± SD (n = 6). Statistical significance: ns, not significant; *p* < 0.05; *p* < 0.01; *p* < 0.001.

As shown in Figure 2A, ovariectomy resulted in marked uterine atrophy, with the OVX group exhibiting a dramatic reduction in uterine size compared with the sham—operated rats. Treatment with raloxifene produced a pronounced recovery of uterine morphology, while EUE, Isf, and particularly their combination showed partial restoration, with visibly thicker uterine horns and improved tissue appearance.

Consistent with these macroscopic changes, Figure 2B shows that uterine weight was significantly reduced in OVX rats (*p* < 0.001 vs. Sham). Raloxifene treatment markedly increased uterine weight, whereas EUE and Isf produced moderate but significant improvements. The combined EUE + Isf treatment further elevated uterine weight and demonstrated a stronger recovery than either agent alone (*p* < 0.01), although remaining slightly lower than the raloxifene group.

Histological examination in Figure 2C further confirmed the estrogen—deficiency phenotype and subsequent recovery. The sham group displayed intact endometrial epithelium, abundant uterine glands, and well—organized stromal architecture. In contrast, the OVX group showed pronounced endometrial thinning, glandular loss, and stromal atrophy. Raloxifene treatment markedly restored glandular density and epithelial height. EUE and Isf each produced moderate histological improvement, while the combined treatment resulted in more prominent recovery, with increased gland numbers and improved endometrial integrity, approaching the structure seen in raloxifene—treated rats.

Bone biomechanical testing (Figure 2D) demonstrated that ovariectomy substantially impaired femoral strength, with maximum load, elastic modulus, and maximum stress all significantly decreased in the OVX group compared with the sham group (*p* < 0.001). Raloxifene markedly reversed these declines. Both EUE and Isf improved biomechanical properties to a moderate degree, whereas the combined EUE + Isf treatment produced the greatest enhancement among the non—raloxifene groups, significantly surpassing the effects of either single agent (*p* < 0.01). These data indicate that EUE and Isf exert additive or synergistic beneficial effects on bone strength in estrogen—deficient rats.

Quantitative histomorphometric analysis of uterine sections was performed to assess endometrial thickness and glandular density. Endometrial thickness was measured at five random points per section, and glandular density was calculated as the number of glands per mm^2^. As shown in Table 3, OVX reduced endometrial thickness by 71% (*p <* 0.001) and glandular density by 68% (*p <* 0.001) compared to sham. Raloxifene treatment restored endometrial thickness to 85% of sham levels. EUE and Isf each produced moderate improvements (45% and 52% of sham, respectively), while the combined EUE + Isf treatment showed significantly greater restoration (68% of sham, *p <* 0.01 vs. either monotherapy). These quantitative data support the histological observations and provide objective measures of uterine protection.

**TABLE 3:** Quantitative histomorphometric analysis of uterine sections.

Group	Endometrial thickness (μm)	% of sham	Glandular density (glands/mm^2^)	% of sham
Sham	245.6 ± 18.3	100%	42.3 ± 4.1	100%
OVX	71.2 ± 9.4^###^	29%	13.5 ± 2.2^###^	32%
OVX + RLX	208.8 ± 15.2^***^	85%	35.2 ± 3.4^***^	83%
OVX + EUE	110.5 ± 12.1^**^	45%	18.9 ± 2.5^**^	45%
OVX + Isf	127.7 ± 11.8^**^	52%	21.5 ± 2.8^**^	51%
OVX + EUE + Isf	167.0 ± 13.5^***,^ ^††^	68%	28.7 ± 3.1^***^ ^,^ ^†^	68%

Data are presented as mean ± SD (n = 6 per group).

RLX: raloxifene; EUE: eucommia ulmoides extract; Isf: Soy isoflavones.

###p < 0.001 vs. Sham group.

** p < 0.01.

*** p < 0.001 vs. OVX, group.

† p < 0.05.

††, p < 0.01 vs. EUE, or Isf alone.

### 3.3 Effects of EUE and Isf on serum bone turnover markers and oxidative stress parameters

**FIGURE 3:**
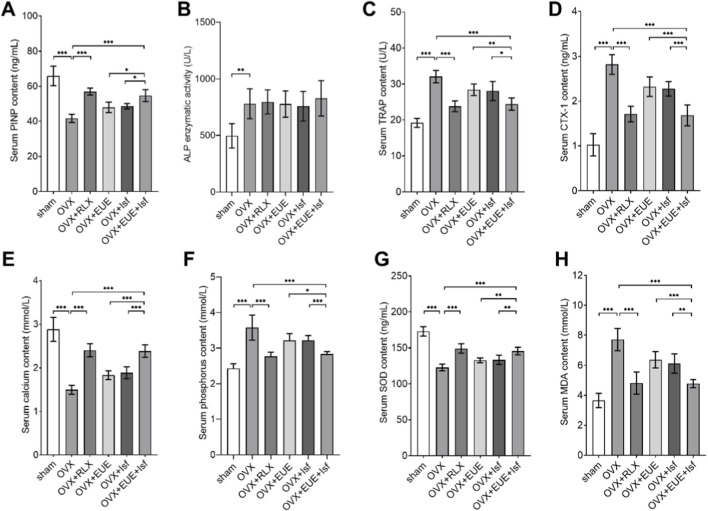
Effects of EUE and Isf on serum bone turnover markers, minerals, and oxidative stress parameters. **(A–D)** Serum bone turnover markers: PINP, ALP, TRAP, and CTX—1, showing OVX—induced imbalance and treatment—related restoration. **(E,F)** Serum calcium and phosphorus levels decreased after OVX and were improved by treatments. **(G,H)** Serum SOD and MDA levels indicating oxidative stress changes and treatment effects. Data are expressed as mean ± SD (n = 6). Statistical significance: *p* < 0.05, *p < 0.01, **p < 0.001.

As shown in Figure 3A, serum PINP levels were significantly reduced in OVX rats compared with the sham group (*p* < 0.001), confirming suppressed bone formation after estrogen deficiency. Raloxifene markedly increased PINP, while EUE and Isf each produced moderate but significant improvements. Combined treatment (EUE + Isf) further elevated PINP levels, exceeding either monotherapy (*p* < 0.05), indicating enhanced osteogenic activity.

Serum ALP activity (Figure 3B) showed a similar pattern. OVX resulted in a significant decline (*p* < 0.01 vs. Sham), whereas raloxifene, EUE, and Isf partially restored ALP levels, with the combined treatment showing the most pronounced improvement.

Bone resorption marker TRAP (Figure 3C) was significantly elevated in the OVX group (*p* < 0.001 vs. Sham). All treatments reduced TRAP concentrations to varying degrees, with the combined EUE + Isf group showing a greater reduction than either agent alone (*p* < 0.01), approaching the effect of raloxifene.

Consistently, serum CTX—1 (Figure 3D) was markedly increased in OVX rats, indicating accelerated bone resorption. Raloxifene significantly lowered CTX—1, whereas EUE, Isf, and the combined treatment each mitigated OVX—induced elevation, with the combination showing the strongest effect among the non—raloxifene groups.

Mineral homeostasis was also altered by ovariectomy. Serum calcium (Figure 3E) and phosphorus (Figure 3F) levels were significantly decreased in OVX rats compared with sham (*p* < 0.001). All treatments improved serum mineral levels, with the EUE + Isf combination showing a more substantial recovery compared with either monotherapy.

Oxidative stress parameters demonstrated a similar trend. Ovariectomy significantly reduced SOD activity (Figure 3G) and increased MDA levels (Figure 3H), reflecting increased systemic oxidative stress. Both EUE and Isf partially reversed these changes, while the combined treatment produced a more pronounced improvement in SOD and a greater reduction in MDA (*p* < 0.01), indicating enhanced antioxidant protection.

### 3.4 Effects of EUE and Isf on trabecular bone microarchitecture in OVX rats

**FIGURE 4:**
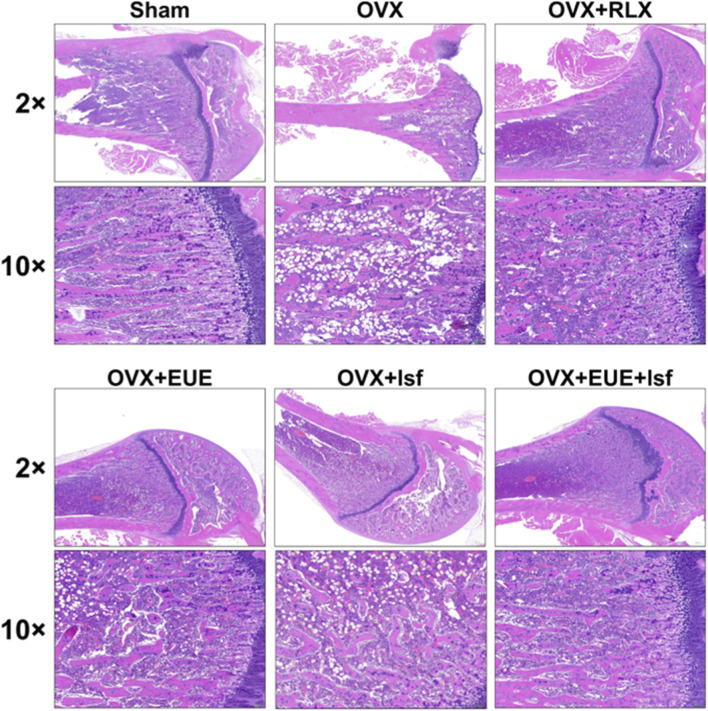
Histological evaluation of trabecular bone in OVX rats following treatment with EUE and Isf.

As shown in Figure 4, H&E staining revealed profound trabecular bone loss in the OVX group compared with the sham controls. In the sham group, both ×2 and ×10 magnifications displayed a dense and continuous trabecular network with well—organized marrow cavities. In contrast, OVX rats exhibited extensive trabecular thinning, marked disruption of structural continuity, enlarged marrow cavities, and reduced trabecular number, consistent with severe estrogen—deficiency–induced bone deterioration.

Raloxifene treatment markedly improved bone microarchitecture, as evidenced by increased trabecular thickness, restored connectivity, and a more compact marrow structure relative to OVX rats. Treatment with EUE or Isf alone resulted in partial improvement, with each treatment showing moderately increased trabecular mass and better structural organization than OVX animals. Notably, the combined EUE + Isf group demonstrated the most substantial recovery among the non—raloxifene groups, with trabecular density, thickness, and continuity visibly enhanced and approaching the morphology observed in the OVX + RLX group. These observations support a stronger restorative effect of the combined treatment on trabecular bone integrity. Quantitative histomorphometric analysis is shown in Table 4. Trabecular bone volume fraction (BV/TV), trabecular thickness (Tb.Th), trabecular number (Tb.N), and trabecular separation (Tb.Sp) were measured in five randomly selected fields per section from six animals per group. As shown in Table 4, OVX significantly reduced BV/TV (by 62%, p < 0.001), Tb.Th (by 38%, p < 0.01), and Tb.N (by 55%, p < 0.001), while increasing Tb. Sp (by 145%, p < 0.001) compared to sham controls. Treatment with EUE or Isf alone partially restored these parameters, while the combined EUE + Isf treatment produced significantly greater improvements: BV/TV increased by 78% compared to OVX (p < 0.001 vs. OVX; p < 0.05 vs. EUE or Isf alone), Tb.Th increased by 42% (p < 0.01), Tb.N increased by 68% (p < 0.001), and Tb. Sp decreased by 52% (p < 0.001). These quantitative data corroborate the qualitative histological observations and demonstrate the superior efficacy of the combination therapy.

**TABLE 4:** Quantitative histomorphometric analysis of trabecular bone in proximal femur.

Group	BV/TV (%)	Tb.Th (μm)	Tb.N (/mm)	Tb.Sp (μm)
Sham	32.4 ± 2.1	85.6 ± 4.2	4.2 ± 0.3	210.5 ± 12.3
OVX	12.3 ± 1.5^***^	53.1 ± 3.8^**^	1.9 ± 0.2^***^	515.8 ± 28.4^***^
OVX + EUE	18.7 ± 1.8^#^	65.4 ± 4.1^#^	2.6 ± 0.3^#^	398.2 ± 22.6^#^
OVX + Isf	19.5 ± 2.0^#^	66.8 ± 3.9^#^	2.7 ± 0.3^#^	387.5 ± 24.1^#^
OVX + EUE + Isf	26.8 ± 2.2^###,^ ^†^	78.9 ± 4.5^##^	3.6 ± 0.4^###,^ ^†^	247.3 ± 18.7^###,^ ^†^
OVX + RLX	28.1 ± 2.4	81.2 ± 4.3	3.8 ± 0.4	231.6 ± 19.2

Data are presented as mean ± SD (n = 6 per group).

BV/TV: bone volume fraction; Tb.Th: trabecular thickness; Tb.N: trabecular number; Tb. Sp: trabecular separation.

*** p < 0.001, p < 0.01 vs. Sham group.

# p < 0.05.

##p < 0.01.

###p < 0.001 vs. OVX, group.

^†^ p < 0.05 vs. EUE, or Isf alone (EUE + Isf vs. monotherapy).

Representative H&E—stained proximal femur sections at ×2 and ×10 magnification showing trabecular morphology in each group. OVX rats exhibited severe trabecular loss and disruption, whereas raloxifene, EUE, Isf, and especially the combined EUE + Isf treatment improved trabecular density and structural integrity to varying degrees.

### 3.5 Effects of EUE and Isf on osteogenic differentiation and cell proliferation *in vitro*

**FIGURE 5:**
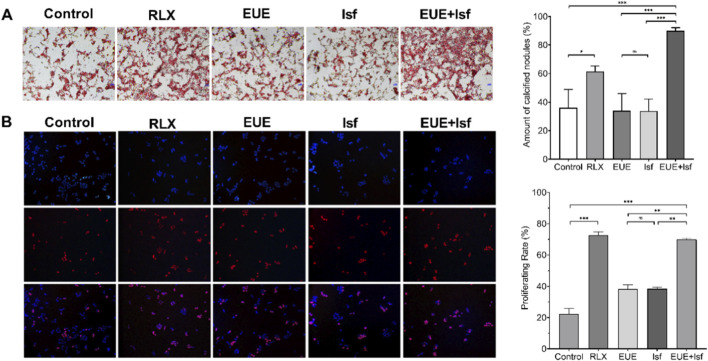
Effects of EUE and Isf on osteogenic mineralization and cell proliferation in MC3T3—E1 cells. **(A)** Alizarin Red S staining showing mineralized nodules after treatment with raloxifene, EUE, Isf, or their combination. Quantification indicates significantly increased mineral deposition, particularly in the combined EUE + Isf group. **(B)** EdU staining of proliferating MC3T3—E1 cells and corresponding quantification. Combined EUE + Isf treatment significantly enhanced proliferation compared with single—agent groups. Data are presented as mean ± SD (n = 3). Statistical significance: ns, not significant; *p* < 0.05; *p < 0.01; **p < 0.001.

As shown in Figure 5A, Alizarin Red S staining demonstrated markedly enhanced mineral deposition following treatment. Compared with the control group, raloxifene substantially increased the number of calcified nodules (*p* < 0.05). EUE and Isf alone produced moderate increases, although the effects were not statistically significant relative to control. Notably, the combined EUE + Isf treatment resulted in the greatest enhancement of mineralization, yielding a significantly higher calcified nodule content than either monotherapy or the control (*p* < 0.001), indicating a synergistic effect on osteogenic differentiation.

Cell proliferation assessed by EdU staining (Figure 5B) showed a similar pattern. Raloxifene markedly increased the proportion of EdU—positive cells compared with the control (*p* < 0.001). EUE and Isf each modestly increased proliferation, with no significant difference between the two treatments. Importantly, the EUE + Isf combination significantly elevated the proliferating rate relative to EUE or Isf alone (*p* < 0.01), approaching the effect observed with raloxifene. These findings indicate that combined treatment promotes osteoblast proliferation more effectively than single—compound treatment.

### 3.6 Effects of EUE and Isf on osteoclast formation and apoptosis in RAW264.7 cells

**FIGURE 6:**
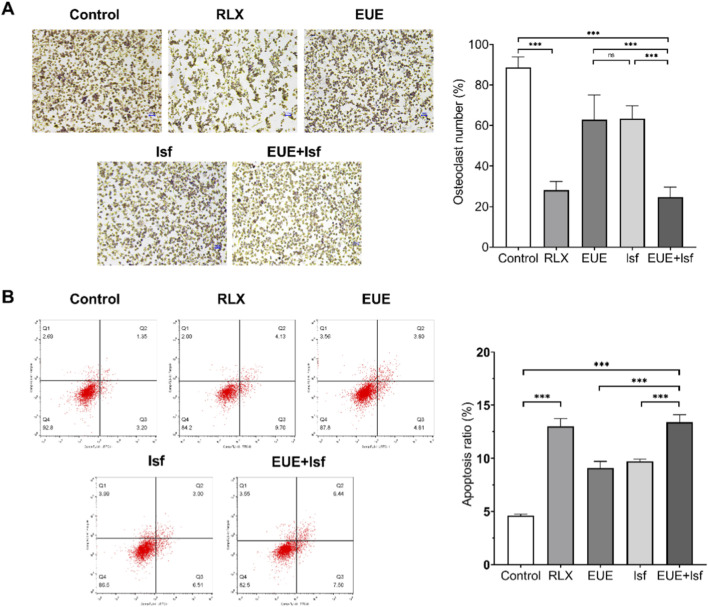
Effects of EUE and Isf on osteoclast differentiation and apoptosis in RAW264.7 cells. **(A)** TRAP staining images and quantification showing RANKL—induced osteoclast formation and its reduction by raloxifene (RLX), EUE, Isf, and combined EUE + Isf treatment. **(B)** Flow cytometry analysis of apoptosis in RAW264.7 cells and corresponding quantification. Combined EUE + Isf treatment showed the strongest pro—apoptotic effect among the non—raloxifene groups. Data are expressed as mean ± SD (n = 3). Statistical significance: ns, not significant; *p* < 0.01; *p* < 0.001.

As shown in Figure 6A, TRAP staining revealed a marked increase in osteoclast formation in the control RANKL—induced group, with numerous TRAP—positive multinucleated cells occupying the culture area. Raloxifene treatment significantly reduced osteoclast number (*p* < 0.001), demonstrating a strong inhibitory effect. EUE and Isf each produced moderate reductions in osteoclast formation, while the combined EUE + Isf treatment further suppressed TRAP—positive cell numbers, resulting in the lowest osteoclast count among the non—raloxifene groups (*p* < 0.001 vs. control; *p* < 0.01 vs. EUE or Isf). The corresponding quantification confirmed this decreasing trend.

Flow cytometric analysis (Figure 6B) demonstrated that RANKL stimulation induced a substantial increase in RAW264.7 apoptosis compared with the control group. Raloxifene treatment led to a significant elevation in apoptotic cells (*p* < 0.001), consistent with its anti—resorptive effect. EUE and Isf produced moderate increases in apoptosis compared with the control, whereas the combined treatment resulted in a further significant rise (*p* < 0.001), with the highest apoptotic proportion observed in the EUE + Isf group. This pattern indicates that combined treatment exerts the strongest pro—apoptotic effect on osteoclast precursors among the test groups.

### 3.7 Effects of EUE and Isf on osteogenic/osteoclastic gene expression and inflammatory cytokines

**FIGURE 7:**
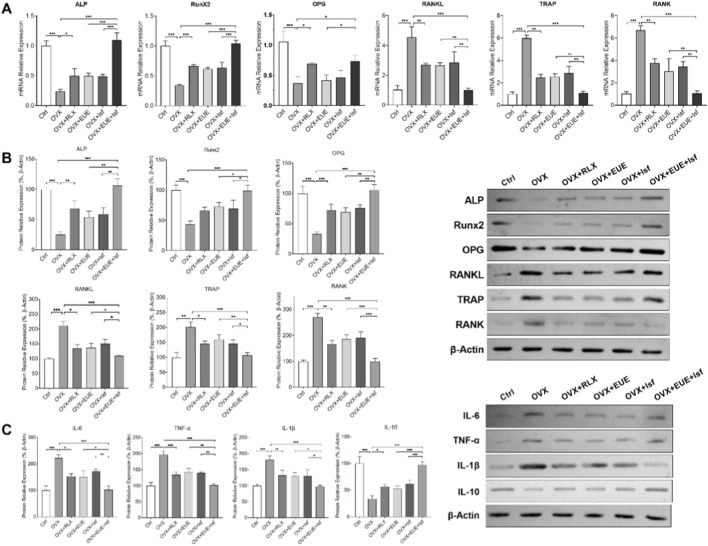
Effects of EUE and Isf on osteogenic and osteoclastic markers and inflammatory cytokines in OVX rats. **(A)** qPCR analysis of ALP, Runx2, OPG, RANKL, TRAP, and RANK mRNA levels in each group. **(B)** Western blot images and quantification of corresponding protein levels showing changes in osteogenic and osteoclast—related markers. **(C)** Western blot analysis of inflammatory cytokines (IL—6, TNF—α, IL—1β, and IL—10). Data are expressed as mean ± SD (n = 3–6). Statistical significance: *p* < 0.05; *p < 0.01; **p < 0.001.

As shown in Figure 7A, qPCR analysis revealed that ovariectomy significantly downregulated the osteogenic markers ALP, Runx2, and OPG (*p* < 0.001 vs. Ctrl), while upregulating RANKL, TRAP, and RANK (*p* < 0.001), indicating impaired bone formation and enhanced bone resorption. Raloxifene largely reversed these changes. Treatment with EUE or Isf partially restored ALP, Runx2, and OPG mRNA expression and reduced RANKL, TRAP, and RANK levels. Notably, the combined EUE + Isf group produced the most prominent improvement, showing significantly higher osteogenic gene expression and lower osteoclast—associated gene expression compared with either monotherapy (*p* < 0.01).

Protein expression trends were consistent with mRNA findings (Figure 7B). Western blot analysis showed reduced ALP, Runx2, and OPG protein levels and elevated RANKL, TRAP, and RANK levels in OVX rats. Raloxifene markedly restored osteogenic proteins and suppressed osteoclast—related proteins. EUE and Isf moderately improved these protein levels, whereas their combination produced a stronger effect, resulting in higher ALP/Runx2/OPG expression and lower RANKL/TRAP/RANK expression than either single treatment, aligning with the quantified bar graphs.

Inflammatory cytokine analysis (Figure 7C) showed that OVX significantly increased IL—6, TNF—α, and IL—1β protein levels (*p* < 0.001), while reducing anti—inflammatory IL—10. Raloxifene reversed these changes, decreasing pro—inflammatory markers and elevating IL—10. EUE and Isf produced moderate anti—inflammatory effects, but the combined treatment showed the most substantial improvements, significantly lowering IL—6, TNF—α, and IL—1β (*p* < 0.01) and increasing IL—10 compared with either agent alone.

## 4 Discussion

This study systematically elucidated the multidimensional protective effects of *Eucommia ulmoides* leaf extract (EUE) and soy isoflavones (Isf) against estrogen deficiency–induced osteoporosis at cellular, tissue, and organismal levels. The most distinctive finding lies in the pronounced synergistic efficacy observed with the combined treatment, which significantly outperformed either agent alone in modulating the balance between osteogenesis and osteoclastogenesis, restoring the bone microenvironment, and regulating inflammation and oxidative stress. These results provide experimental evidence supporting the use of plant—based combinatorial therapies for menopausal osteoporosis and offer new insights into the development of multi—component, precision—targeted phytotherapeutic strategies.

The core pathophysiology of postmenopausal osteoporosis involves impaired osteoblast function, excessive osteoclast activation, and systemic dysregulation of the immune–oxidative stress network (Fischer and Haffner-Luntzer, 2022; He et al., 2023; Yao et al., 2025). Although current treatments such as selective estrogen receptor modulators and bisphosphonates partially improve bone density, long—term use is often associated with adverse effects and fails to address the complex pathological dimensions of the bone microenvironment (Fink et al., 2019; Miyoshi et al., 2021; Motlani et al., 2023). Our findings demonstrate that EUE and Isf exert multifaceted actions: they enhance bone formation, restrain bone resorption, improve trabecular architecture, strengthen antioxidant capacity, suppress proinflammatory cytokine production, and ultimately improve bone mechanical properties. This broad, network—based regulatory profile aligns with the emerging paradigm in osteoporosis research that emphasizes bone microenvironment reconstruction rather than single—target modulation, underscoring the theoretical innovation of our approach.

**FIGURE 8:**
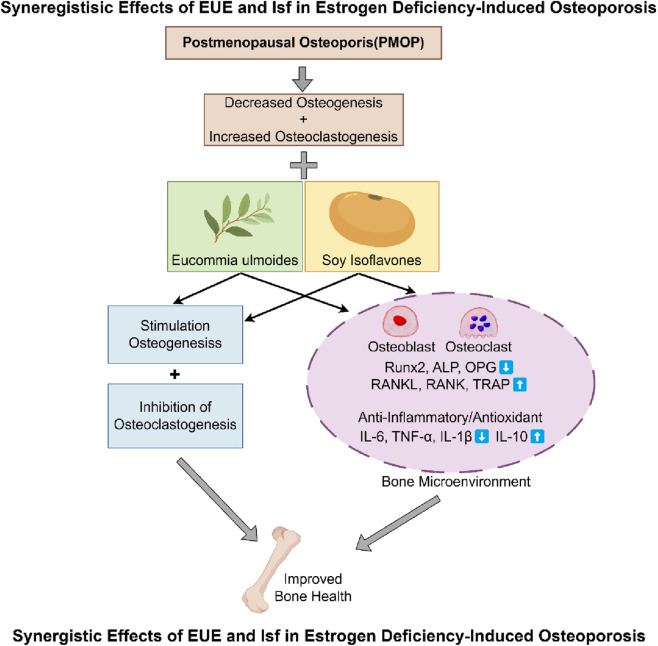
Proposed mechanism underlying the synergistic protective effects of Eucommia ulmoides extract (EUE) and soy isoflavones (Isf) in estrogen—deficiency–induced osteoporosis.

Importantly, the integration of these actions can be visualized through the mechanistic model illustrated in Figure 8, which summarizes the complementary roles of EUE and Isf in estrogen deficiency–induced bone deterioration. The figure highlights how estrogen withdrawal leads to reduced osteogenesis, heightened osteoclastogenesis, and increased inflammatory and oxidative stress burden. EUE and Isf counteract these pathological processes from distinct yet converging directions—EUE primarily providing anti—inflammatory and antioxidant support, while Isf contributes phytoestrogenic stimulation of osteoblast activity (Harahap and Suliburska, 2021; Huang L. et al., 2021). Together, they synchronously promote osteogenesis, inhibit osteoclast differentiation, and remodel the bone microenvironment toward a state conducive to maintaining bone mass and structural integrity. This mechanistic visualization further reinforces the synergy observed in our experimental findings and provides a conceptual framework for plant—derived combination therapies.

An important mechanistic consideration raised by our findings relates to the source of estrogenic activity in EUE. While our EUE preparation was standardized to chlorogenic acid (a phenolic acid), chlorogenic acid itself is not a classical phytoestrogen and does not directly bind to estrogen receptors (Ding et al., 2024). The estrogen—like effects observed in this study—including partial uterine weight restoration and modulation of bone turnover—are therefore likely attributable to other constituents present in the extract, particularly lignans (such as pinoresinol, medioresinol, and syringaresinol) and iridoids (such as aucubin and geniposidic acid), which have documented phytoestrogenic properties (Ong and Tan, 2007; Ding et al., 2024). Recent metabolomic studies have demonstrated that following oral administration, EUE components undergo extensive biotransformation, yielding metabolites with enhanced estrogenic activity (Ding et al., 2024). For example, lignan glycosides are converted by gut microbiota to enterolignans (enterodiol and enterolactone), which structurally resemble endogenous estrogens and can bind to estrogen receptors. This prodrug—like mechanism explains how extracts containing non—estrogenic precursors can exert phytoestrogenic effects *in vivo*. Thus, the estrogenic activity we observed likely results from the collective action of multiple EUE components and their metabolites, rather than chlorogenic acid alone. This understanding resolves the apparent contradiction and highlights the importance of considering both the extract composition and its *in vivo* metabolism when interpreting phytoestrogenic effects.

In addition to their skeletal effects, the combined EUE and Isf treatment also exhibited estrogenic activity in reproductive tissues, as evidenced by the partial restoration of uterine weight and endometrial integrity. While this may suggest potential benefits for urogenital health in postmenopausal women, it also raises important safety considerations regarding the risk of endometrial hyperplasia—a critical concern for any therapy with estrogen—like properties (Cline et al., 2002; Mareti et al., 2019). Notably, the degree of uterine weight increase with the combined treatment (approximately 65% of sham levels) was substantially lower than that observed with raloxifene, a selective estrogen receptor modulator with established endometrial safety, and far below the supraphysiological stimulation associated with unopposed estrogen therapy. Histological examination revealed preserved endometrial architecture with no evidence of hyperplasia, glandular crowding, or cytological atypia in any treatment group. This favorable safety profile may be attributed to the selective estrogen receptor modulation properties of both EUE components (particularly lignans) and soy isoflavones, which preferentially activate ERβ over ERα—the receptor subtype primarily responsible for endometrial proliferation (Mareti et al., 2019). Nevertheless, long—term studies specifically designed to assess endometrial safety, including evaluation of proliferative markers (e.g., Ki—67) and estrogen—responsive genes, are warranted before clinical translation. These findings underscore the importance of balancing therapeutic efficacy with endometrial safety in the development of phytoestrogen—based therapies for postmenopausal osteoporosis.

A crucial conceptual advance from this study is the recognition that low—dose plant—derived bioactives may exhibit complementary pharmacological windows, resulting in synergistic amplification of osteogenic, anti—resorptive, anti—inflammatory, and antioxidative effects. The biphasic dose–response patterns observed in vitro—where low concentrations stimulate cell proliferation while high concentrations suppress viability—indicate that these phytochemicals possess non—linear pharmacodynamics that challenge traditional dose–effect paradigms. The enhanced efficacy of the combined low—dose regimen suggests that optimal therapeutic outcomes may depend more on rational component matching and dose structuring than on increasing the amount of a single agent. This concept provides a valuable direction for the modernization and rational design of botanical combination formulations.

The observed biphasic dose-response *in vitro*, characterized by cytotoxicity at higher concentrations, further emphasizes the importance of therapeutic window optimization. High-dose cytotoxicity of plant extracts often arises from non-specific effects, such as membrane perturbation, mitochondrial dysfunction, or excessive reactive oxygen species generation (Ekor, 2014). For EUE, concentrations exceeding 300 ng/mL may overwhelm cellular antioxidant capacity, triggering oxidative stress and apoptosis. For Isf, high concentrations can inhibit topoisomerase II and induce DNA damage—mechanisms distinct from their estrogenic effects at lower doses (Markovits et al., 1989). These findings highlight that therapeutic benefit is not simply a function of dose escalation; rather, identifying the optimal concentration range is critical.

At the molecular level, the combined treatment more effectively enhanced the expression of osteogenic markers while suppressing genes associated with osteoclast activation. These changes were accompanied by reductions in IL—6, TNF—α, and IL—1β, alongside restoration of IL—10. This cross—dimensional improvement—spanning bone formation, bone resorption, inflammation, and oxidative stress—suggests potential mechanistic complementarity between the two plant extracts. Chlorogenic acid, the primary marker compound in EUE, has been shown to activate the Nrf2 antioxidant pathway and inhibit NF—κB signaling, thereby reducing oxidative stress and inflammation—both critical contributors to bone loss in estrogen deficiency ——(Guo et al., 2022; Shen et al., 2024; Liang et al., 2025). Lignans from EUE, particularly pinoresinol and syringaresinol, are metabolized to enterolignans that can activate estrogen receptor signaling, explaining the observed effects on uterine weight and OPG expression. Soy isoflavones (genistein, daidzein) are well—established ERβ—selective agonists that directly modulate osteoblast and osteoclast activity through regulation of Runx2 and RANKL (Sampey et al., 2011). The convergence of these complementary mechanisms—antioxidant, anti—inflammatory, and phytoestrogenic—provides a rational basis for investigating their combined effects on the RANKL/RANK/OPG axis and downstream transcription factors controlling bone remodeling (Zhu et al., 2021; Wan et al., 2025). This phytochemical rationale strengthens the mechanistic framework of our study and supports the multi—target approach to postmenopausal osteoporosis therapy.

Several limitations of this study should be acknowledged. First, while our EUE preparation was standardized to chlorogenic acid, it remains a complex mixture of multiple bioactive constituents. The specific compounds responsible for the observed effects, and their potential synergistic interactions, require further investigation through bioactivity—guided fractionation and metabolomic studies (You et al., 2023; Ding et al., 2024). Second, the *in vivo* study employed a single dose level for each treatment; dose—response studies with multiple dose combinations would be necessary to identify the optimal therapeutic ratio. Third, although we provide evidence for modulation of key regulatory pathways, definitive pathway validation using specific inhibitors or genetically modified models is needed to establish causality (Wan et al., 2025). Fourth, although we observed consistent changes in key regulators of bone remodeling (Runx2, ALP, OPG, RANKL) and inflammatory cytokines, our study did not perform direct pathway validation experiments (e.g., using specific pathway inhibitors or genetically modified models) to establish causality. While the observed associations support the involvement of these pathways, definitive proof would require intervention studies that block or activate specific signaling nodes. Future research employing tools such as NF—κB inhibitors, RANKL—neutralizing antibodies, or OPG knockout models would provide more conclusive evidence for the causal roles of these pathways in mediating the therapeutic effects of EUE and Isf. Fifth, our study focused on bone and uterine endpoints; assessment of other estrogen—sensitive tissues (e.g., mammary gland, cardiovascular system) would provide a more complete safety profile. Sixth, while we performed quantitative histomorphometry, micro—CT analysis would provide more comprehensive three—dimensional assessment of trabecular microarchitecture. Finally, the translational relevance of our findings to humans requires confirmation in clinical studies, as rodent models do not fully replicate human menopause and bone remodeling dynamics (Liao et al., 2003). Despite these limitations, our study provides robust evidence for the therapeutic potential of EUE + Isf combination and establishes a foundation for future mechanistic and translational research.

In conclusion, this study is the first to systematically demonstrate that a low—dose synergistic regimen combining *Eucommia ulmoides* extract and soy isoflavones substantially mitigates bone microenvironmental imbalance and bone loss under estrogen—deficient conditions. Through comprehensive evaluation of bone cell function, trabecular structure, oxidative stress, and inflammatory responses, our findings highlight the multidimensional regulatory potential of this combination therapy. The proposed mechanistic model (Figure 8) integrates these observations, providing a coherent biological framework for the synergistic actions of the two plant extracts. These results support the candidacy of this phytocompound combination as a multi—target botanical intervention and offer a theoretical foundation for advancing compound herbal therapeutics toward precision—oriented clinical applications for menopausal osteoporosis.

## 5 Conclusion

This study demonstrates that *Eucommia ulmoides* leaf extract and soy isoflavones, when administered in a low—dose synergistic regimen, exert comprehensive protective effects against estrogen deficiency–induced osteoporosis. The combination therapy simultaneously enhances osteogenic activity, suppresses osteoclastogenesis, mitigates inflammatory and oxidative stress responses, and improves trabecular structure and bone mechanical strength. These multidimensional regulatory effects reflect a complementary interaction between the two plant—derived bioactives and highlight the therapeutic potential of rationally designed phytocompound combinations for restoring bone microenvironment homeostasis.

Importantly, this work identifies a cooperative biological window in which low—dose natural compounds can achieve superior efficacy compared with monotherapy, offering a conceptual basis for advancing network—oriented, multi—target botanical therapeutics. The mechanistic framework proposed in this study provides new hypotheses for future investigations into phytochemical synergy using multi—omics, lineage tracing, and bone–immune interaction analyses. Collectively, the findings support the development of EUE–Isf combination therapy as a promising, multi—target intervention strategy for the precision management of menopausal osteoporosis.

## Data Availability

The original contributions presented in the study are included in the article/Supplementary Material, further inquiries can be directed to the corresponding author.
